# Nitric Oxide Synthase and Breast Cancer: Role of TIMP-1 in NO-mediated Akt Activation

**DOI:** 10.1371/journal.pone.0044081

**Published:** 2012-09-05

**Authors:** Lisa A. Ridnour, Kimberly M. Barasch, Alisha N. Windhausen, Tiffany H. Dorsey, Michael M. Lizardo, Harris G. Yfantis, Dong H. Lee, Christopher H. Switzer, Robert Y. S. Cheng, Julie L. Heinecke, Ernst Brueggemann, Harry B. Hines, Chand Khanna, Sharon A. Glynn, Stefan Ambs, David A. Wink

**Affiliations:** 1 Radiation Biology Branch, National Cancer Institute, Bethesda, Maryland, United States of America; 2 Laboratory of Human Carcinogenesis, National Cancer Institute, Bethesda, Maryland, United States of America; 3 Tumor and Metastasis Biology Section, Pediatric Oncology Branch, National Cancer Institute, Bethesda, Maryland, United States of America; 4 Pathology and Laboratory Medicine, Baltimore Veterans Affairs Medical Center, Baltimore, Maryland, United States of America; 5 USAMRIID, Fort Detrick, Maryland, United States of America; Case Western Reserve University, United States of America

## Abstract

Prediction of therapeutic response and cancer patient survival can be improved by the identification of molecular markers including tumor Akt status. A direct correlation between NOS2 expression and elevated Akt phosphorylation status has been observed in breast tumors. Tissue inhibitor matrix metalloproteinase-1 (TIMP-1) has been proposed to exert oncogenic properties through CD63 cell surface receptor pathway initiation of pro-survival PI3k/Akt signaling. We employed immunohistochemistry to examine the influence of TIMP-1 on the functional relationship between NOS2 and phosphorylated Akt in breast tumors and found that NOS2-associated Akt phosphorylation was significantly increased in tumors expressing high TIMP-1, indicating that TIMP-1 may further enhance NO-induced Akt pathway activation. Moreover, TIMP-1 silencing by antisense technology blocked NO-induced PI3k/Akt/BAD phosphorylation in cultured MDA-MB-231 human breast cancer cells. TIMP-1 protein nitration and TIMP-1/CD63 co-immunoprecipitation was observed at NO concentrations that induced PI3k/Akt/BAD pro-survival signaling. In the survival analysis, elevated tumor TIMP-1 predicted poor patient survival. This association appears to be mainly restricted to tumors with high NOS2 protein. In contrast, TIMP-1 did not predict poor survival in patient tumors with low NOS2 expression. In summary, our findings suggest that tumors with high TIMP-1 and NOS2 behave more aggressively by mechanisms that favor Akt pathway activation.

## Introduction

Breast cancer is a heterogeneous disease defined by distinct tumor phenotypes that vary in prognosis and response to therapeutic agents and remains the second leading cause of cancer related deaths among women in the United States [1]. Although standard therapeutics have improved the outlook and quality of life, 16% of women with regional lesions and 76% of women with metastatic disease continue to lose their fight against breast cancer within the first five years of diagnosis [1]. Clinical management of breast cancer currently employs diagnostic patient evaluations aimed at designing more “personalized” therapeutic modalities including tamoxifen for the treatment of estrogen receptor positive (ER+) disease, or trastuzumab for human epidermal growth factor receptor-2 (HER2) positive disease. Recent evidence suggests that this personalized approach will continue as numerous phase II and phase III clinical trials are examining the roles of new targeted therapies in the management of breast cancer. Toward this end, alterations in the PI3k/Akt and RAS signaling pathways have been identified as frequent and important events in both the establishment and maintenance of malignancies [2]. The Akt family of serine/threonine kinases demonstrates oncogenic behavior by mediating survival of various cell types through promotion of cell cycle progression and inhibition of apoptosis [3]. Moreover, increased Akt phosphorylation has correlated with poor disease-free survival of breast cancer patients [4], [5].

Activation of Akt signaling can occur through many pathways. One such pathway involves tissue inhibitor matrix metalloproteinase-1 (TIMP-1) [6]–[8]. TIMPs are natural inhibitors against the proteolytic activity of matrix metalloproteinases (MMPs), the balance of which governs matrix remodeling during normal physiology and pathogenesis. Other studies have discovered more complex, MMP-independent roles for TIMPs in the regulation of cell proliferation, differentiation, angiogenesis, and survival during tumor progression [7]. From a clinical perspective, TIMP-1 is correlated with poor prognosis in several cancers including breast and colorectal tumors, as well as lymphoma and non-small cell carcinoma of the lung [8]. TIMP-1 activates pro-survival signaling through its binding to the tetraspanin CD63/integrin ß1 complex at the cell surface of breast epithelial cells [9]. The intracellular cytoplasmic tail of CD63 interacts with FAK and activates PI3 kinase and regulates cell adhesion, motility, and survival [7]. FAK has a central role in organizing cell structure and signaling, and is over expressed and active in tumor cells [10], [11]. Thus, studies have implicated the TIMP-1/CD63/integrin complex in the regulation of FAK/PI3k/Akt and downstream pro-survival signaling in breast cancer [7].

Clinical findings support the potential use of TIMP-1 as a predictive biomarker for patient therapeutic response and cancer survival [8]. Studies have correlated high TIMP-1 with breast and colorectal tumor resistance to hormone therapy, cyclophosphamide/methotrexate/5-fluorouracil, cyclophosphamide/epirubicin/5-fluorouracil, cyclophosphamide/adriamycin/5- fluorouracil, or single agent adriamycin [12], [13]. In addition, two independent clinical trials have revealed an involvement of TIMP-1 in resistance to hormone therapy in estrogen receptor/progesterone receptor positive patients with metastatic breast cancer [14], [15]. In support of these observations, TIMP-1 was found to reduce tumor cell sensitivity to chemotherapeutic drug treatment of cells grown in culture [16]. This finding likely involves the induction of DNA damage and P53 activation by anti-cancer drugs, which culminates in tumor cell apoptosis that is inhibited by TIMP-1-mediated activation of FAK/PI3k/Akt signaling [7], [17]. Indeed, TIMP-1 suppression enhanced chemotherapeutic sensitivity and tumor cell apoptosis by inhibiting Akt signaling, while its overexpression promoted tumor aggressiveness of MDA-MB-231 breast cancer cells [16], [18].

Chronic inflammation also promotes tumor development and cancer progression [19]. Nitric oxide (NO) generated by the inducible NO synthase (NOS2) isoform is an important pro-inflammatory mediator linking chronic inflammation with cancer progression [20], [21]. Elevated NOS2 expression has been identified in precancerous lesions [19] and numerous studies suggest a potential use of NOS2 as a predictive cancer biomarker with high NOS2 tumor expression being associated with poor patient survival in several types of cancer including melanoma, prostate, and breast [22]–[27]. More recently, NOS2 expression was found to correlate directly with traditional predictive markers of survival including tumor grade, mutant P53, and tumor microvascularization, and predicted poor breast cancer specific survival at five and ten years in women with ER negative disease [28]. Also, a pro-inflammatory microenvironment associated with tumor derived NOS2 directly correlated with tumor pAkt status and downstream signaling including p-caspase-9 and pBAD in the same breast tumors [29]. Because TIMP-1 mediates Akt signaling and is also regulated by NO [30]–[33] we explored the relationship between TIMP-l, NOS2, and Akt phosphorylation signaling in the same breast tumors.

## Results

### Tumor IHC and Patient Survival Analyses

**Figure 1:**
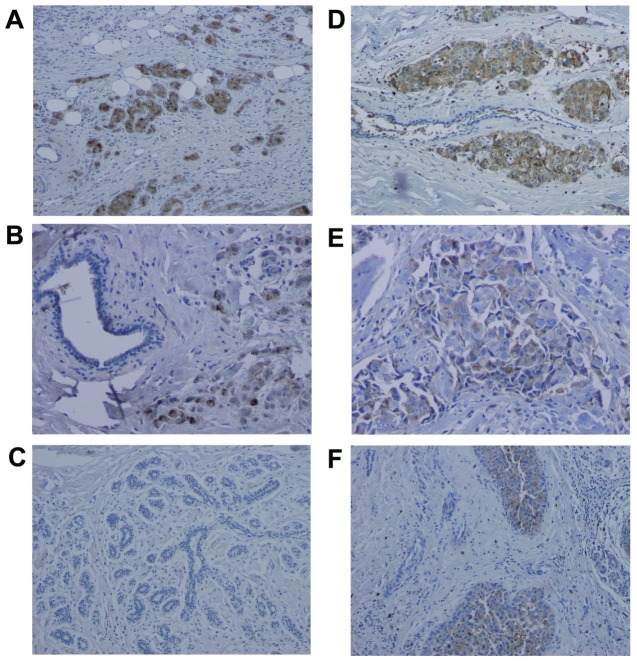
Expression of TIMP-1 and CD63 in human breast tumors. Shown is immunohistochemical staining for TIMP-1 (A–C) and CD63 (D–F). (A) Strong staining of TIMP-1 is shown in the tumor epithelial cells. The surrounding tissue consisting of stromal cells and adipocytes is negative for TIMP-1. (B) Moderate staining of TIMP-1 in tumor epithelial cells is shown in the lower center and to the right. Normal epithelial cells are negative for TIMP-1 (upper left corner). (C) Normal epithelial cells in surrounding non-tumor tissue of an invasive breast tumor are negative for TIMP-1. (D) Strong staining of CD63 in the tumor epithelium. The surrounding tissue consisting of stromal cells and a benign breast duct is mostly negative for CD63. (E) Moderate staining of CD63 in tumor epithelial cells (center). (F) Moderate to strong staining of CD63 in tumor epithelial cells (lower and upper center). Normal epithelial cells to the left and stromal cells are negative for CD63. Magnification: 100× for A, C, D, F; 200× for B and E. Counterstain hematoxylin.

**Figure 2:**
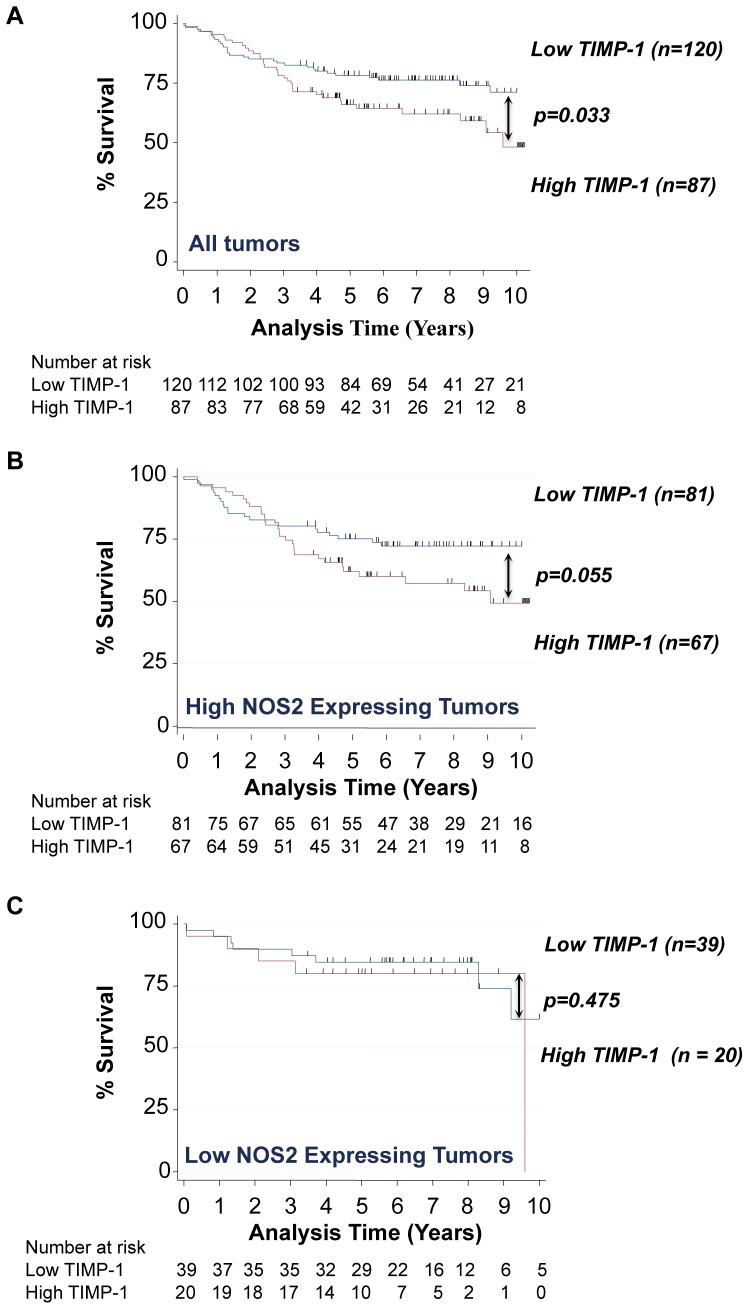
TIMP-1 predicts poor breast cancer survival in patients whose tumors express elevated NOS2 but not in those with low NOS2 expression. Kaplan-Meier survival analysis demonstrating reduced breast cancer specific survival in A) patients whose tumors express elevated TIMP-1 protein and B) a similar trend when stratified for tumors with high TIMP-1 and high NOS2 protein levels. C) In contrast, TIMP-1 did not predict poor survival of patients with low NOS2 tumor expression.

Prueitt et al. previously found that tumor derived NOS2 expression correlated with increased Akt phosphorylation, which was supported by experiments showing NO activation of Akt and downstream signaling in cultured cells [29]. Herein, we examined the influence of TIMP-1 on the relationship between tumor NOS2 and pAkt-(ser473) [29] using immunohistochemical analysis. To accomplish this, the expression of both TIMP-1 and its cell surface receptor CD63 were examined in 217 surgically resected non-metastatic breast carcinomas whose tumor NOS2 and Akt phosphorylation status was reported in two previous publications [28], [29]. TIMP-1 and CD63 exhibited moderate to strong expression in 42% and 41%, respectively, of the breast tumors (Figure 1A–C and D–F, respectively). Distribution of histology grading and TNM staging [28], [29] are shown in Table 1: TIMP-1 had no effect on tumor grade or stage while elevated CD63 tumor expression correlated with high tumor grade. NOS2 expression in these tumors has been reported [28], [29] and was found to predict poor survival of ER negative breast cancer patients [28]. While elevated tumor TIMP-1 also predicted poor breast cancer specific survival in this study (Figure 2A), further stratification of the breast tumors into those with high and those with low NOS2 expression led to the observation that the association of TIMP-1 with poor survival seemed to be mainly restricted to tumors with high NOS2 expression (Figure 2B). In contrast, TIMP-1 did not predict poor survival in patients with low tumor NOS2 expression (Figure 2C). Together, these results suggest that the effects of aberrantly high TIMP-1 and NOS2 on breast cancer outcome may be mechanistically interrelated.

**Table 1:** Marker expression by disease stage and grade.

Disease Characteristic	NOS2 Low		NOS2 High		?^2^ test
TNM Stage	n	%	n	%	p-value
Low (≤II)	53	84	131	79	
High (III/IV)	10	16	34	21	0.418
Grade 1	12	20	22	14	
Grade 2	29	48	45	29	
Grade 3	19	32	88	57	0.004

Disease Characteristic	TIMP-1 Low		TIMP-1 High		?^2^ test
TNM Stage	n	%	n	%	p-value
Low (≤II)	94	82	70	79	
High (III/IV)	20	18	19	21	0.495
Grade 1	21	19	10	12	
Grade 2	32	30	32	38	
Grade 3	55	51	43	50	0.263

Disease Characteristic	CD63 Low		CD63 High		?^2^ test
TNM Stage	n	%	n	%	p-value
Low (≤II)	86	81	60	79	
High (III/IV)	20	19	16	21	0.715
Grade 1	22	22	6	8	
Grade 2	29	29	31	40	
Grade 3	50	49	40	52	0.027

Next, we examined correlations between the expression of TIMP-1, NOS2, as well as other markers of disease progression that were previously described in these patient tumors [29]. Using the Spearman correlation test, we found that tumor TIMP-1 expression correlated positively with NOS2, and with the phosphorylation status of Akt substrates p-caspase-9 and nuclear pBAD, as well as tumor associated MMP-1 and MMP-9 as summarized in Table 2. For most markers, this association was restricted to ER negative tumors. These results suggest that the functional relationship between TIMP-1, NOS2, and Akt pathway activation is perhaps more significant in the ER-negative than the ER-positive disease.

**Table 2:** TIMP-1 Correlates with NOS2, p-caspase-9, nuclear pBAD, MMP-1, and MMP-9.

TIMP-1/Marker	Spearman's rho			p-value		
	All	ER(−)	ER(+)	All	ER(−)	ER(+)
NOS2	0.09	0.22	0.01	0.155	0.04	0.889
p-caspase-9	0.13	0.34	0.03	0.055	0.001	0.762
pBAD nuc	0.09	0.31	−0.05	0.168	0.002	0.508
MMP-1	0.19	0.23	0.14	0.006	0.034	0.112
MMP-9	0.30	0.37	0.28	<0.001	<0.001	0.001
TIMP-1*_normal epithelia_*	0.44	0.34	0.51	<0.001	0.008	<0.001

TIMP-1 has been shown to mediate Akt activation and pro-survival signaling [7], [17]. A significant relationship between NOS2 expression and Akt phosphorylation (NOS2/pAkt) was reported for these breast tumors [29]. Thus, we studied the modifying effects of TIMP-1 on this pathway and used logistic regression analysis to calculate adjusted odds ratios (OR) as a method to describe the strength of an association between variables after adjustment for possible confounders. We employed this statistical test to examine the impact of TIMP-1 expression on the NOS2/pAkt correlation reported by Prueitt et al. [29]. After adjustment for age at diagnosis, tumor ER status, and TNM stage, an OR of 4.5 described the significant relationship between NOS2 and phosphorylated Akt at ser473 in these tumors [29]. After stratification of tumors into those with low and high TIMP-1 expression, a remarkably increased OR was observed for the relationship between NOS2 and phosphorylated Akt-(ser473) in tumors with high TIMP-1 (OR = 12.7) whereas the OR was reduced to 2.5 in tumors with low TIMP-1 (Table 3). These data are consistent with the mechanistic model that high NOS2 expression leads to a significantly increased activation of Akt in breast tumors in the presence of elevated TIMP-1. In contrast, if TIMP-1 is low or absent, activation of Akt, as judged by ser473 phosphorylation, is reduced. Thus, the data show that elevated TIMP-1 augments NO-mediated phosphorylation of Akt at ser473 in these breast tumors (Table 3). Similar data were obtained when we analyzed the effect of TIMP-1 on the NOS2/pAkt-(thr308) relationship in the same tumors; generally, however, the modifying effects of TIMP-1 appeared to be weaker in this analysis (Table 3). While CD63 expression did not significantly modify the correlation between NOS2/Akt phosphorylation at ser473, the analysis of the Akt phosphorylation status at Akt thr308 indicated that high CD63 has modifying effects similar to those of TIMP-1 and it enhanced that of NOS2/pAkt-(thr308) by approximately two-fold. These results suggest that TIMP-1 augments the effects of NOS2 expression on Akt phosphorylation, which in turn may provide an opportunity for therapeutic interventions in patients whose tumors express elevated levels of TIMP-1, NOS2, and have increased phosphorylated Akt.

**Table 3:** TIMP-1 augments NOS2/pAkt association in breast tumors.

	Unadjusted			Adjusted*	
NOS2/pAkt(ser473)	Spearman's rho	p-value	OR	95% CI	p-value
All Tumors (n = 248)	0.31	<0.001	4.49	2.08–9.71	<0.01
Low TIMP-1 (n = 125)	0.21	0.017	2.50	0.88–7.08	0.083
High TIMP-1 (n = 92)	0.51	<0.001	12.7	3.14–51.1	<0.001
Low CD63 (n = 116)	0.34	<0.001	4.41	1.61–12.01	0.004
High CD63 (n = 81)	0.31	0.006	4.59	0.79–26.3	0.088

NOS2/pAkt(thr308)	Spearman's rho	p-value	OR	95% CI	p-value
All Tumors (n = 248)	0.20	0.002	2.27	2.08–9.71	<0.01
Low Timp-1 (n = 125)	0.17	0.054	2.10	0.87–5.08	0.096
High Timp-1 (n = 92)	0.26	0.011	3.08	1.05–9.04	0.04
Low CD63 (n = 116)	0.23	0.012	2.87	1.12–7.36	0.028
High CD63 (n = 81)	0.34	0.003	4.60	1.23–17.2	0.023

* adjusted for age at diagnosis, ER status and TNM stage.

**Figure 3:**
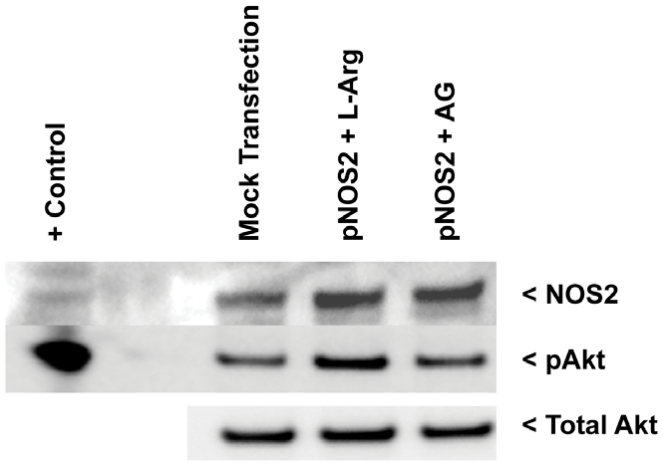
Transfection of NOS2 expression plamid enhances pAkt. NOS2 overexpressing MDA-MB-231 breast cancer cells treated with L-Arginine show increased pAkt. Treatment of the same NOS2 transfected cells with the NOS2 inhibitor aminoguanidine (AG) show no increase in pAkt.

To verify Akt activation by NOS2-derived NO, we overexpressed NOS2 in breast cancer cells then treated the cells with the NOS substrate L-Arginine or the NOS2 inhibitor aminoguanidine (AG). Estrogen receptor negative MDA-MB-231 breast cancer cells were used because of the association between the expression levels of NOS2 and TIMP-1 observed in ER negative breast cancer patients as shown in Table 2. When compared to the mock control, L-Arg treated NOS2 overexpressing cells exhibit enhanced Akt phosphorylation, which is diminished by the NOS2 inhibitor aminoguanidine as shown in Figure 3. These results demonstrate Akt activation by NOS2-derived NO.

**Figure 4:**
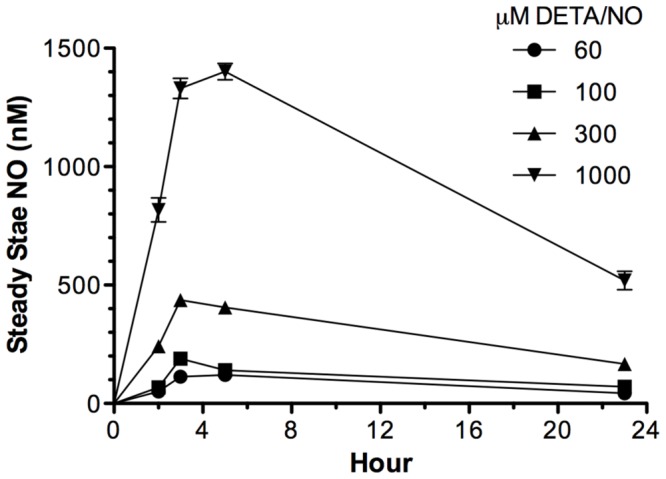
Temporal steady state NO flux generated by the NO Donor DETA/NO. Steady state nM NO levels distributed evenly throughout cell culture media as a function of NO donor (DETA/NO) concentration measured through 24 hr.

Nitric oxide is a diffusible, gaseous molecule. Our laboratory and others have demonstrated that NO effects are concentration, spatially, and temporally dependent (20, 21, 28), thus we employed the slow releasing NO donor DETA/NO (T*_1/2_*∼20 hr at 37°C) for the remaining cell culture experiments to gain insight on the steady state NO levels that modulate target molecules. Steady state NO plotted as a function of time is shown in Figure 4, which demonstrates the steady state nM NO levels diffused throughout the media with respect to defined donor concentrations.

### TIMP-1 Suppression Abolishes NO-induced pAkt in Breast Cancer Cells

**Figure 5:**
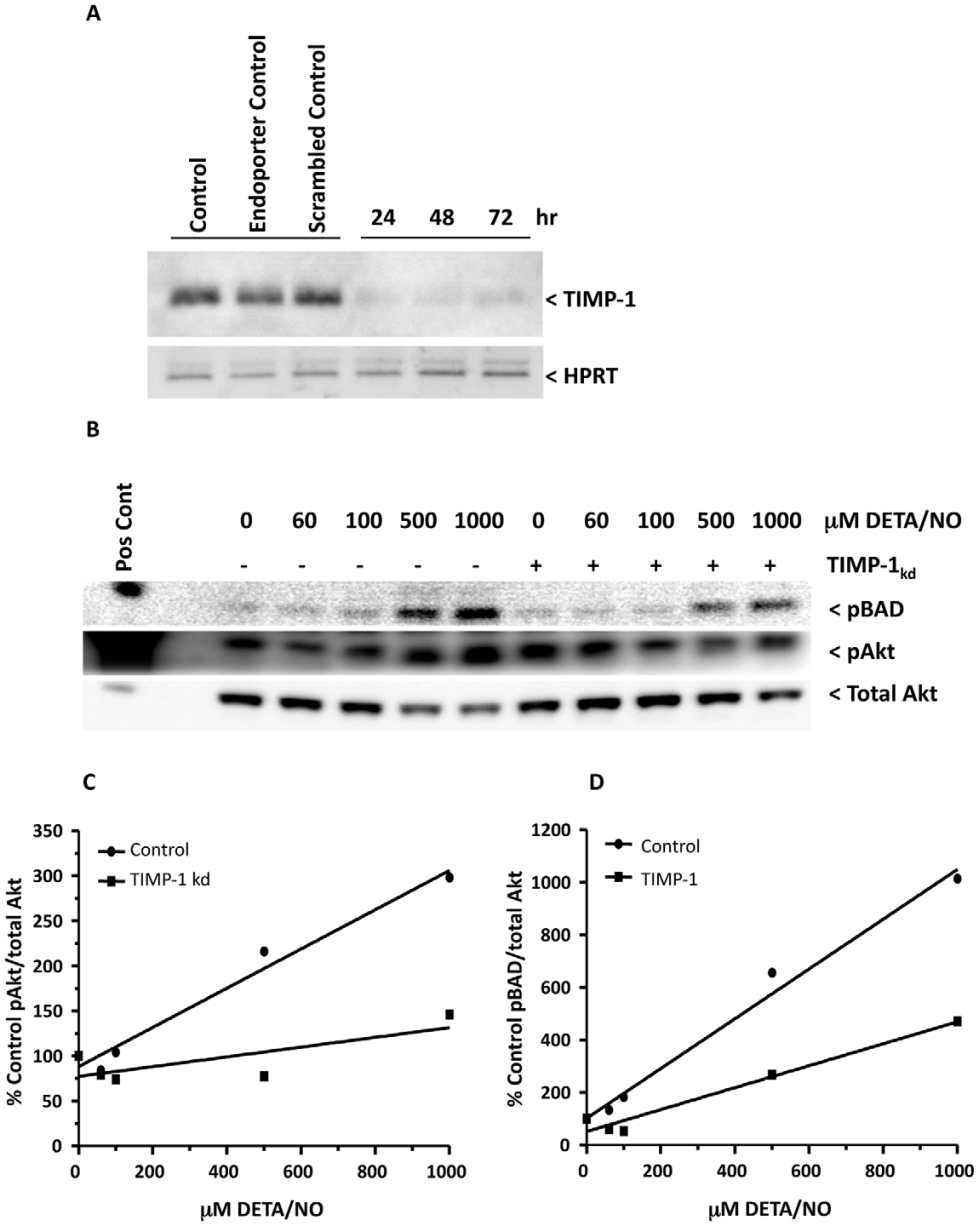
Western blots demonstrate the requirement of TIMP-1 for NO-induced Akt phosphorylation in MDA-MB-231 breast cancer cells. A) Suppression of TIMP-1 protein translation using anti-sense TIMP-1 morpholino through 72 hr. B) TIMP-1 suppression (TIMP-1_kd_) abolishes NO-induced Akt phosphorylation and reduces the extent of NO-mediated phosphorylation of Akt downstream pro-apoptotic target BAD. The cells were incubated with TIMP-1 morpholino as described in the methods section, serum starved, and then treated with increasing concentrations of DETA/NO for 24 hr. The western blots shown were stripped and reblotted for total Akt are representative of two independent experiments. C–D) Densitometry measurements of pAkt (C) and pBAD (D) relative to total Akt loading control were plotted vs. DETA/NO concentration. Linear regression pAkt/total Akt: control, r^2^ = 0.974, y-intercept = 90.40+/−10.35, slope = 0.2187+/−0.021 p = 0.0018; TIMP-1 kd, r^2^ = 0.573, y-intercept = 76.04+/−14.61, slope = 0.0583+/−0.029, p = 0.1381. Linear regression pBAD/total Akt: control, r^2^ = 0.988, y-intercept = 91.45+/−29.29, slope = 0.9053+/−0.058, p = 0.0006; TIMP-1 kd, r^2^ = 0.953, y-intercept = 44.60+/−24.30, slope = 0.3747+/−0.048, p = 0.0027.

Logistic regression analysis of IHC results for the breast tumors showed that the association between NOS2 and pAkt-(ser473) was strongest in tumors with elevated TIMP-1 protein but reduced in those with low TIMP-1 expression. Therefore, we examined the impact of experimental TIMP-1 knockdown (TIMP-1_kd_) on NO-mediated Akt phosphorylation in the MDA-MB-231 cells. TIMP-1 protein translation was suppressed using an anti-sense morpholino construct designed to specifically inhibit protein translation of TIMP-1 transcript, which reduced the levels of TIMP-1 protein secreted by MDA-MB-231 breast cancer cells through at least 72 hr (Figure 5A). Next, the effect of TIMP-1 protein suppression on NO-induced Akt phosphorylation was examined. Control morpholino and TIMP-1 knockdown MDA-MB-231 cells were serum starved overnight and then treated with DETA/NO for 24 hr. Nitric oxide exposure of cells pretreated with control morpholino show increased pAkt-(ser473) at 500 and 1000 µM DETA/NO, which was abolished in the absence of TIMP-1 (Figure 5B). Similarly, NO-induced phosphorylation of the pro-apoptotic molecule and downstream Akt target BAD was observed at 500 and 1000 µM DETA/NO and was also suppressed in the absence of TIMP-1 (Figure 5B). Linear regression analyses can be used to describe the relationship between two variables. Therefore, linear regression analyses of densitometric values of pAkt/total Akt (Figure 5C), or pBAD/total Akt (Figure 5D), plotted vs. DETA/NO concentration was performed and are summarized in Figure 5 legend. These results demonstrate a role of TIMP-1 during NO-induced pro-survival signaling that is mediated, at least in part, through Akt activation in MDA-MB-231 cells grown in culture, and are consistent with the observations in breast tumors demonstrating enhanced NOS2/pAkt-(ser473) in breast tumors expressing high TIMP-1 protein (Table 3).

### TIMP-1 Protein Expression in Breast Cancer Cells Treated with NO Donor

**Figure 6:**
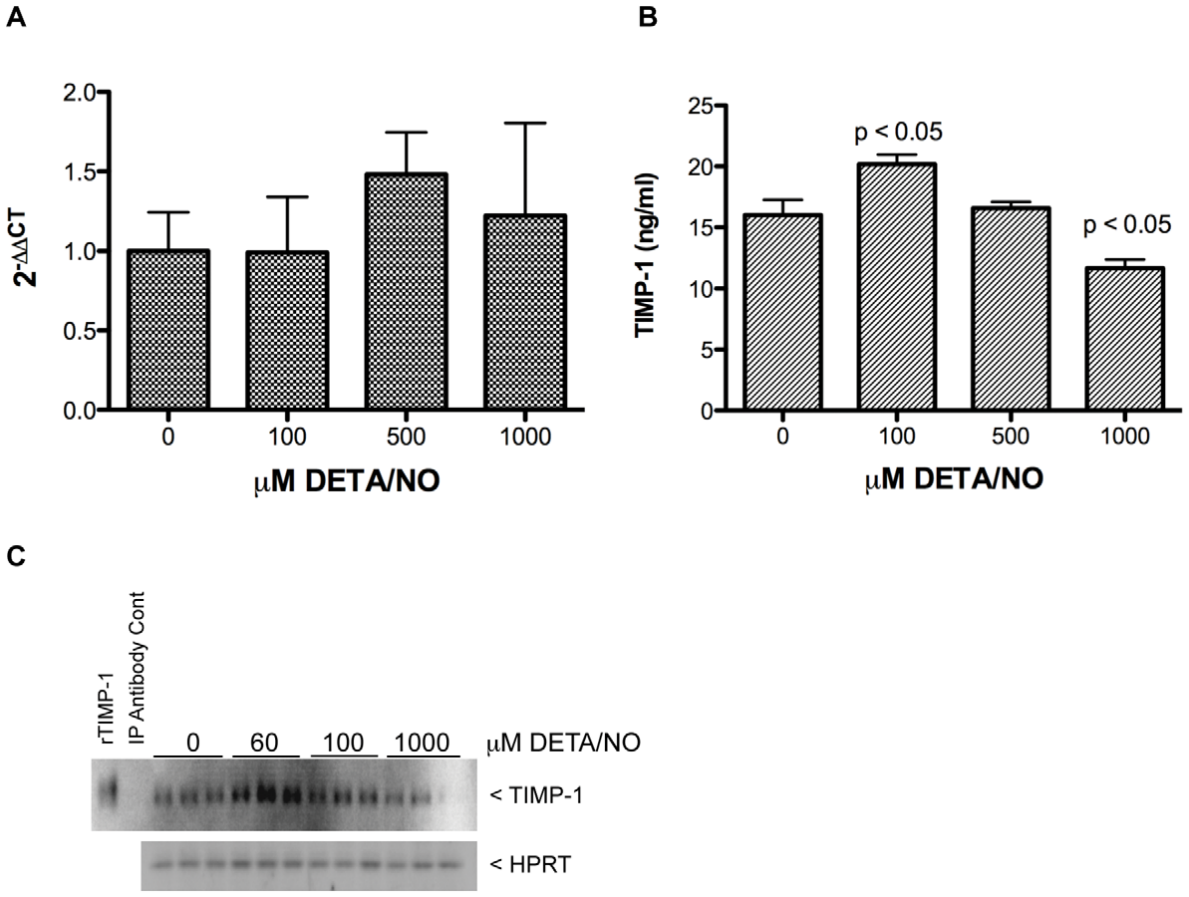
NO modulation of TIMP-1 protein levels. A) NO does not significantly effect steady state TIMP-1 mRNA as shown by real time PCR (n = 5). B) NO modulates TIMP-1 protein in the media from MDA-MB-231 breast cancer cells as shown by ELISA assay. The results are presented as mean+/−SEM of n = 4 samples. C) NO modulates TIMP-1 protein in the media from human fibroblasts as shown by western blot.

Nitric oxide regulation of TIMP-1 has been described in cancer cells, as well as macrophages, and fibroblasts, which are key components of tumor stroma [30]–[33]. We examined the effect of NO on TIMP-1 mRNA and protein levels in MDA-MB-231 breast cancer cells. While steady state TIMP-1 mRNA did not change significantly (Figure 6A), modest but significant changes in TIMP-1 protein in the media are shown in Figure 6B following treatment of MDA-MB-231 cells with DETA/NO. Tumor stroma may actively participate in cancer progression by secretion of cytokines and growth factors that are beneficial to the tumor. Because fibroblasts are primary components of tumor stroma and have been shown to secrete pro-tumorigenic factors within the tumor microenvironment, we examined the effect of NO on TIMP-1 protein in the media of fibroblasts. Figure 6C shows increased levels of TIMP-1 in the media of fibroblast cells treated with 60–100 µM DETA/NO. These results are consistent with MDA-MB-231 TIMP-1 protein modulation in response to NO and suggest that TIMP-1 protein can be regulated in different cell types within the tumor microenvironment by gradient levels of NO.

### TIMP-1 Co-localization with Cell Surface Receptor CD63

**Figure 7:**
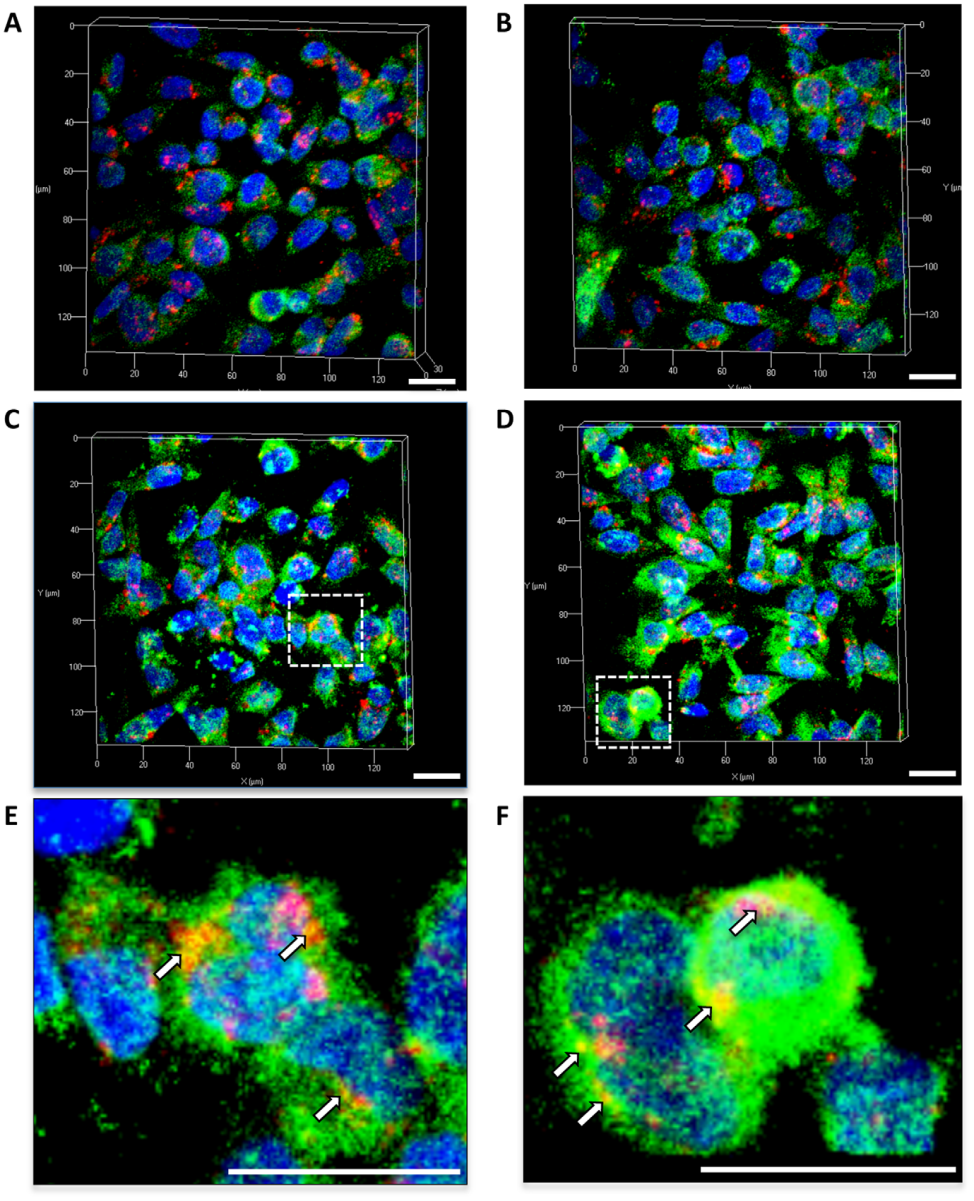
Confocal image microscopy shows effects of NO on CD63 and TIMP-1 protein co-localization in MDA-MB-231 breast cancer cells treated with DETA/NO. CD63 (red fluorescence) and TIMP-1 (green fluorescence) in MDA-MB-231 breast cancer cells treated for 24 hr with the NO donor DETA/NO at the following concentrations; A) control, B) 100 µM, C) 500 µM, D) 1000 µM. Panels E and F are the enlarged areas circled in panels C and D, respectively, which show areas of CD63 and TIMP-1 protein co-localization (yellow/orange fluorescence). Cellular nuclei are visualized as blue fluorescence (DAPI). Scale bar = 20 µm for all panels.

The interaction of TIMP-1 with its cell surface receptor CD63 has been shown to initiate pro-survival signaling through Akt activation [9]. To further investigate the mechanism associated with TIMP-1 enhanced Akt activation by higher steady state concentrations of NO, we examined the effects of NO on TIMP-1 co-localization with CD63. MDA-MB-231 cells were exposed to increasing concentrations of DETA/NO and examined for CD63 protein expression (red fluorescence), TIMP-1 protein expression (green fluorescence), and TIMP-1/CD63 co-localization (orange/yellow fluorescence) by confocal microscopy. Figure 7A demonstrates CD63 and TIMP-1 fluorescence in untreated control cells. Fluorescence intensity associated with 100 µM DETA/NO (Figure 7B) showed little to no effect. However, increased TIMP-1 fluorescence (green) was observed in response to 500–1000 µM DETA/NO (Figure 7C–D). CD63 fluorescence (red) appeared consistent at all NO concentrations. Co-localization of TIMP-1 and CD63 is shown as orange/yellow fluorescence, which appears to be increased at 500–1000 µM DETA/NO (Figure 7 C–D and inset Figure 7E–F) when compared to the untreated control (Figure 7A).

### TIMP-1 Nitration by NO

**Figure 8:**
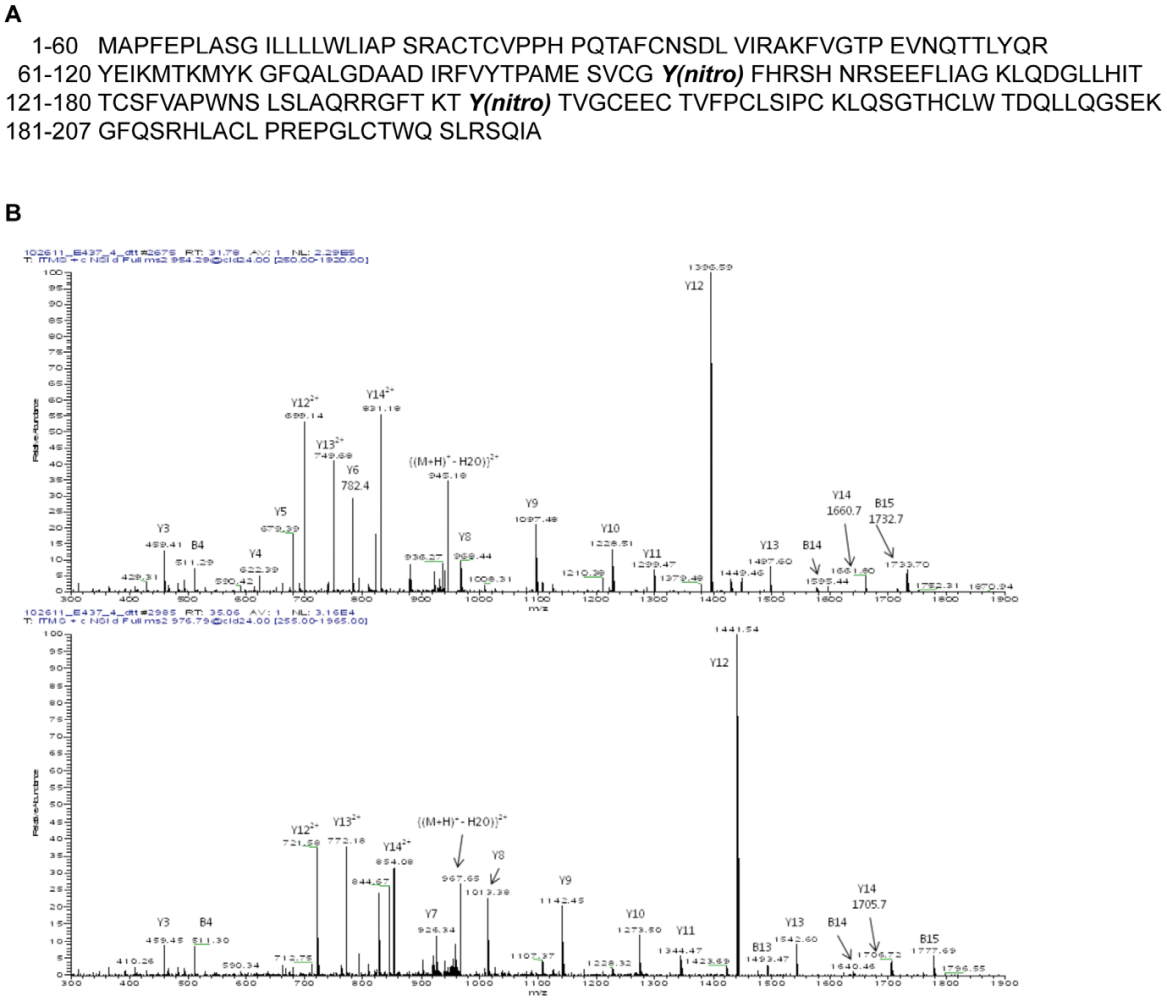
Mass spectrum showing nitration of two tyrosine residues (Y95, Y143) in human recombinant TIMP-1 protein treated with 100, 500, or 1000 µM DETA/NO. A) amino acid sequence of human recombinant TIMP-1 protein (NCBI Reference Sequence: NP_003245.1) and location of tyrosine nitration (Y95, Y143). B) representative mass spectrum of a nitrated peptide from human TIMP-1, illustrating the mass shift of 45 daltons that accompanied nitration.

Protein nitration by NO can impact signaling pathways. To examine this possibility, human recombinant TIMP-1 protein was treated (24 hr) with or without DETA/NO in 24-well cell culture plates to mimic cell culture growth conditions. Figure 8A shows the amino acid sequence for human TIMP-1 protein (NCBI Reference Sequence: NP_003245.1). Five µg aliquots of TIMP-1 were exposed to increasing DETA/NO concentrations (0, 100, 500, or 1000 µM). After chemical treatment, exposed and control proteins were digested with trypsin to generate peptides for analysis. Nitration was not observed in the untreated control TIMP-1 protein. However, tyrosine nitration was detectable at Y143 in native human TIMP-1 following treatment with 100 µM DETA/NO. Initially, nitrated Y143 was detected as part of a tryptic dipeptide joined by a disulphide bond. This was confirmed by DTT treatment of the reacted, native, trypsinized sample, which produced two peptides, one nitrated and one unaffected by DETA/NO. Figure 8B contains a representative mass spectrum of a nitrated peptide from human TIMP-1, illustrating the mass shift of 45 daltons that accompanied nitration. It was also observed that Y143 was the only residue affected by 100 µM DETA/NO concentration. In addition to tyrosine nitration at Y143, recombinant TIMP-1 protein exposed to 500–1000 µM DETA/NO concentrations yielded a second tyrosine nitration event at Y95. No additional nitrated residues were detectable at these higher DETANO concentrations. Both tyrosines in human TIMP-1 are located near key cysteine residues (C93 and C147) that are involved in separate disulfide bonds (C24–C93 and C26–C147) within the native protein. Although these tyrosines in human TIMP-1 were nitrated, the % nitration for these specific tyrosines was low. Using reconstructed ion chromatograms for appropriate unaffected and nitrated peptides (data not shown), it was found that the ratio of peak areas for a given peptide versus its corresponding nitrated peptide was high, indicating that most tyrosines at these locations were largely unaffected by the DETA/NO treatment in a substantial number of protein molecules. The ratios of peak areas at each concentration calculated nitration % yields of 3.9% (100 µM), 8.9% (500 µM), and 8.4% (1000 µM DETA/NO), which translates to 195–455 ng of nitrated recombinant TIMP-1 protein per 5 µg aliquot.

**Figure 9:**
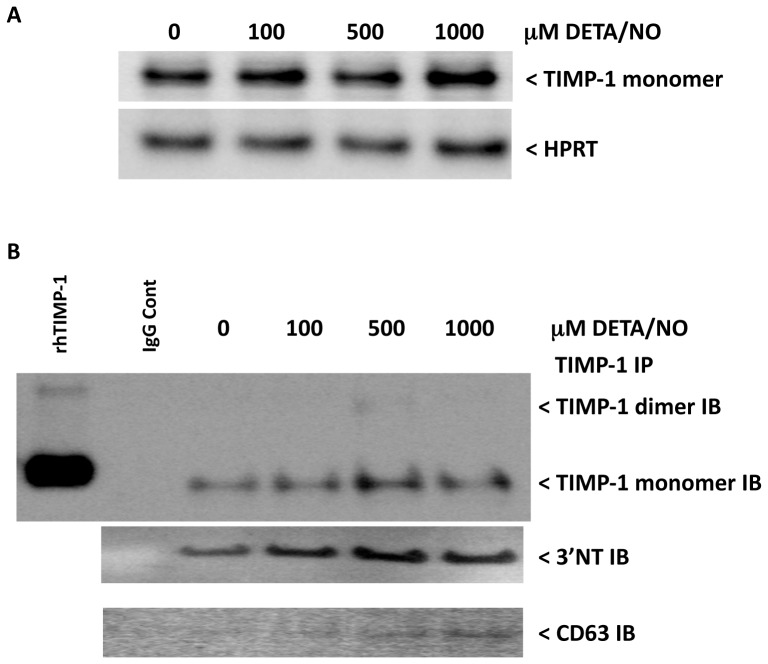
TIMP-1 immunoprecipitation of DETA/NO treated MDA-MB-231 cell lysates. A) Western blot showing TIMP-1 protein expression in the lysates of DETA/NO treated MDA-MB-231 breast cancer cells. B) TIMP-1 immunoprecipitated from DETA/NO treated MDA-MB-231 cells and immunoblotted for 3′NT and CD63 demonstrates enhanced TIMP-1 tyrosine nitration and CD63 co-immunoprecipitation associated with DETA/NO exposure.

Next, we examined TIMP-1 nitration in the cell lysates of MDA-MB-231 cells treated with DETA/NO. This was accomplished by immunoprecipitation of TIMP-1 from equal protein aliquots of the cell lysates, then blotting for TIMP-1 and 3′NT. Figure 9A shows TIMP-1 levels in the DETA/NO treated MDA-MB-231 cell lysates, while immunoprecipitated TIMP-1 blotted for TIMP-1 and 3′NT is shown in Figure 9B. Interestingly, a basal level of 3′NT was observed in immunoprecipitated TIMP-1 control cell lysates, which increased with respect to DETA/NO treatment. It should also be noted that the 3′NT signal appeared higher on the blot (∼60 kD) and migrated consistent with TIMP-1 dimer (Figure 9B). In addition, enhanced co-immunoprecipitation of CD63 (53 kD) with TIMP-1 in lysates treated with 500–1000 µM DETA/NO was observed. These results demonstrate enhanced tyrosine nitration of TIMP-1 protein, which co-immunoprecipitated with the cell surface receptor CD63 in DETA/NO treated MDA-MB-231 cells.

**Figure 10:**
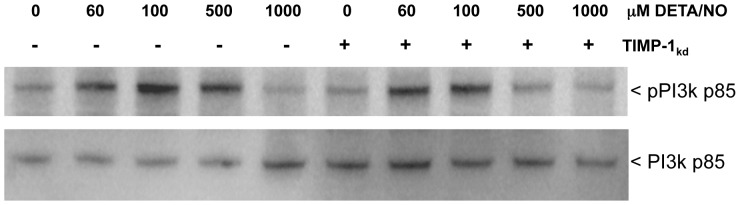
DETA/NO induced PI3k p85 phosphorylation is suppressed in TIMP-1 knockdown cells.

**Figure 11:**
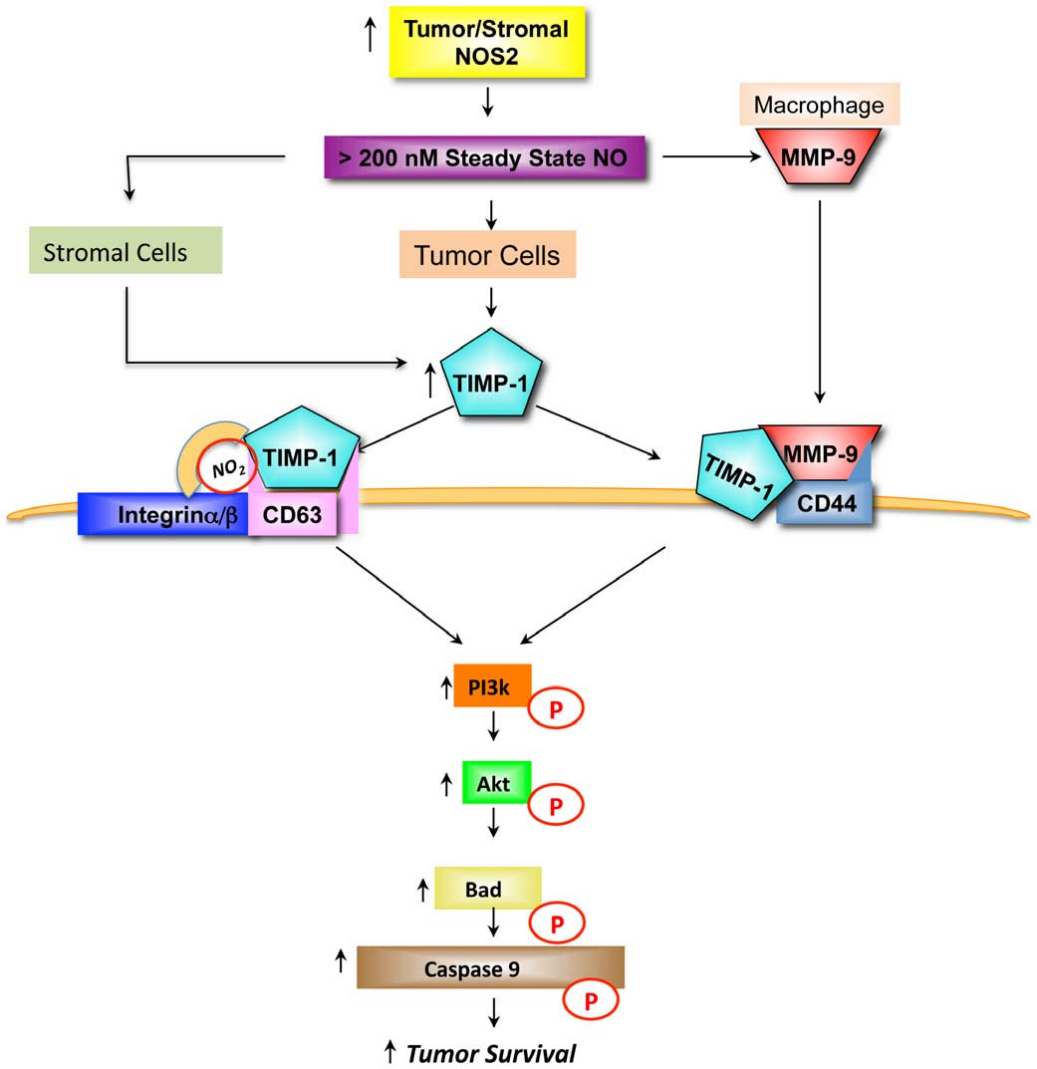
NOS2/NO promotes TIMP-1-dependent pro-survival signaling through Akt activation in the breast tumor microenvironment.

Chirco et al have presented a model where TIMP-1 initiates pro-survival pathways by binding to CD63, which initiates FAK and downstream PI3k/Akt/BAD signaling [7]. Toward this end, we examined the effects of NO on PI3k activation in control and TIMP-1 knockdown cells. Figure 10 demonstrates NO-induced phosphorylation of PI3k p85 at 60, 100, and 500 µM DETA/NO, which was diminished in TIMP-1 knockdown cells. Together these results suggest that TIMP-1 nitration may, at least in part, be an important event in NO-induced PI3k/Akt/BAD pro-survival signaling and that elevated TIMP-1/NOS2/pAkt may be useful predictors of breast cancer patient survival as summarized in Figure 11.

## Discussion

Tissue inhibitor matrix metalloproteinase-1 has been proposed to exert oncogenic properties through binding of CD63 cell surface receptor and subsequent activation of pro-survival PI3k/Akt signaling [7]. This report identifies for the first time a functional association between the inflammatory mediator NOS2 and TIMP-1 during Akt pathway activation in human breast tumors. Stratification of tumors by high versus low TIMP-1 expression showed augmented NOS2 association with Akt-(ser473) phosphorylation in tumors with high TIMP-1 protein expression. In contrast, tumors with low TIMP-1 demonstrated a reduced NOS2 association with pAkt-(ser473). This finding was supported by TIMP-1 knockdown studies in cultured human breast cancer cells showing diminished NO-induced PI3k/Akt/BAD phosphorylation by TIMP-1 silencing. Moreover, TIMP-1 protein nitration and TIMP-1/CD63 co-immunoprecipitation was observed at NO concentrations that induced PI3k/Akt/BAD pro-survival signaling. CD63 is also involved in protein trafficking and may facilitate TIMP-1 internalization [7]; this proposed model may explain, in part, the increased intracellular levels of TIMP-1 observed at 500–1000 µM DETA/NO by confocal microscopy (Figure 7) as well as the decreased levels of TIMP-1 in the media of 1000 µM DETA/NO treated cells (Figure 6B), and further supports a NO-mediated TIMP-1/CD63 protein-protein interaction. While NO increased TIMP-1 in the media at lower (60–100 µM DETA/NO) donor concentrations and may involve enhanced protein translation [34], in our hands pro-survival Akt activation occurred at higher levels of NO concurrent with TIMP-1 nitration. TIMP-1 predicted poor patient survival in a Kaplan-Meier survival analysis, which was restricted to tumors with high NOS2 protein. In contrast, TIMP-1 did not predict poor survival in patient tumors with low NOS2 expression. These results suggest that tumors with high TIMP-1 and NOS2 may behave more aggressively by mechanisms that favor Akt pathway activation. While several studies have linked TIMP-1 with Akt activation and pro-survival signaling *in vitro*, this is the first study, to the best of our knowledge, correlating TIMP-1-dependent Akt activation in human breast tumors with poor patient survival. As many chemotherapeutics target cancer cells by inducing apoptosis, this novel observation may be of particular relevance to clinical findings that show a functional relationship between high TIMP-1, resistance to apoptosis, and tumor drug resistance [12]–[15]. Moreover, patients with NOS2/TIMP-1/pAkt marker signature may benefit by drug therapies that target these pathways including chemopreventive dithiolethiones, which are clinically available [35], [36].

The application of NOS2 as a predictive biomarker and drug target is rapidly emerging as an attractive candidate in cancer therapy [22]–[29], [37]–[39]. In the same breast tumors reported herein, Glynn et al. have recently identified NOS2 as a predictor of basal-like disease in ER negative tumors [28]. NOS2 was associated with additional cancer biomarkers in these tumors, including pAkt, down stream targets p-caspase-9, and pBAD, as well as the stem cell marker CD44 in ER negative tumors [28], [29]. Interestingly, CD44 also correlates with increased aggressiveness and resistance to radiation therapy and chemotherapeutics (reviewed in [39]) and TIMP-1 binding of the proMMP-9/CD44 complex has been shown to mediate cell survival through Akt activation [40]. Herein, TIMP-1 is shown to correlate with NOS2, pAkt, p-caspase-9, and nuclear pBAD in ER negative tumors (Table 2) and TIMP-1 suppression reduced NO-induced phosphorylation of PI3k/Akt as well as the downstream pro-apoptotic target BAD in MDA-MB-231 cells grown in culture, which further supports a role for this pathway in breast tumors. Gene expression profiling has identified TIMP-1 within a basal marker subcluster (mesenchymal subcluster) in breast cancer cell lines including MDA-MB-231 cells [41]. Thus, TIMP-1 constitutes an additional marker of tumor aggressiveness and poor disease outcome for which NOS2 correlates in these breast tumors. A functional relationship between NOS2 and TIMP-1 during PI3k/Akt activation and pro-survival signaling is summarized in the schematic shown in Figure 11.

It is well documented that NO mediates both tumor promoting and tumor suppressive effects and that these outcomes are NO-concentration and -temporally dependent (reviewed in 20,21, and 39]. NOS2 generates higher levels of NO, and appears to be a better cancer biomarker than the other NOS isoforms, NOS1 and/or NOS3, which are both constitutively regulated and generate low NO flux. NOS2 can produce a range of concentrations of NO, depending upon the specific stimulant (i.e. IFN, IL-1, TNF, LPS) [42]. In addition to the Akt signaling pattern shown herein, threshold levels of NO can promote tumorigenesis by targeting other key mediators of cancer progression and angiogenesis including phosphorylation of p53 (1000 nM steady state), stabilization of HIF-1alpha (300–800 nM steady state), protein translational machinery and proliferation signaling pathways (100 nM Steady state) [29], [34], [43]. At lower levels (2–50 nM steady state), NO regulates extracellular matrix proteins including thrombospondin-1 (TSP-1), MMP-9, and TIMP-1 in endothelial cells and macrophages [30], [31], [44]. Together, these studies embody an evolving mechanistic model where distinct levels of NO influence and regulate specific signaling mediators within the tumor microenvironment that in turn promote tumor survival and progression, which may include TIMP-1 and Akt signaling as shown in this study.

A specific NO flux can distinguish key signaling pathways involved in tumor progression that are activated by mechanisms involving direct target interaction with NO at low NO flux or RNS at high levels of NO [20], [21]. For example, cGMP-dependent stimulation of ERK phosphorylation mediated by 10–50 nM steady state NO occurs in MCF-7 cells [43]. NO levels above 50 nM were found to be Ras-dependent but cGMP-independent [34]. The involvement of RNS at the NO flux (≥400 nM steady state NO) described herein led to TIMP-1 tyrosine nitration at Y95 and Y143. Indeed, tyrosine nitrosation rearranges to form 3′NT [45], [46], which may be an important event for TIMP-1/CD63 receptor PI3k/Akt/BAD signaling by NO. Also, TIMP-1 nitration has recently been reported in human macrophages treated with LPS, which reduced its MMP inhibitory function [47] and may provide a model favoring MMP-independent TIMP-1/CD63-mediated signaling under nitrosative conditions. Nitrosative stress promotes other anti-apoptotic pathways; Kim et al. showed that NO flux associated with IFN/TNF treatment inhibited apoptosis of HT-29 human colon cancer cells through nitrosation of caspase-9 [48]. A high NO flux >1 µM steady state NO produced by LPS-induced Toll-like receptor activation mediated nitrosation and down regulation of apoptosis signal regulating kinase-1 (ASK1) and subsequent suppression of caspase-3 activity in LPS treated myeloid leukemia cells [49]; this high level of NO involves formation of RNS [42].

The pathways described above may not be unique to cancer cells but may also function in part as an epithelial or fibroblast cellular response to inflammation. Indeed, an exploratory statistical analysis demonstrated a correlation between NOS2 and TIMP-1 in normal appearing breast epithelial cells (χ^2^ = 8.41, p = 0.038) in tumor surrounding breast tissue, suggesting that low levels of NO may induce TIMP-1 release from normal epithelial cells in a manner analogous to that shown for fibroblasts and cancer cells grown in culture (Figure 6). TIMP-1 associated Akt activation may also provide a mechanism for normal epithelial cell protection during inflammation. TIMP-1 has been shown to protect epithelial cells from intrinsic and extrinsic cell death through FAK/PI3k/Akt signaling [17], [50] and an oncogenic function of TIMP-1 has been postulated to arise through such pro-survival signaling [17]. More recently, TIMP-1 deficiency promoted enhanced ischemia reperfusion injury in a mouse model that was associated with reduced pAkt/Bcl2 and enhanced caspase-3 activation [51]. TIMP-1 is also a biomarker of cellular senescence, which serves as a protective barrier against carcinogenesis [52]. Interestingly, NO was recently associated with senescence in inflammatory bowl disease [53]. This may constitute an interesting yin/yang role of NO/TIMP-1 in non-cancerous cells where secreted TIMP-1 activates pro-survival signaling but not proliferation of the senescing cell, as TIMP-1 can mediate both pro- and anti-proliferative responses [54]. Moreover, studies have shown that premature senescent fibroblasts secrete factors that conferred tumor promotion of neighboring cells [55]. Thus, it is plausible that TIMP-1 secreted from fibroblasts may impose an oncogenic activity on neighboring epithelial cells and supports the hypothesis that gradient levels of NO could regulate TIMP-1 within a tumor microenvironment, which may have a key role during cancer promotion and progression. The observation of NOS2 expression in precancerous lesions [19] suggests that these responses could be facilitated by NOS2 expressing cells, as the cellular proximity to the NOS2 enzyme dictates the concentration of NO that cells experience and subsequent pathways that are elicited by gradient concentrations of NO [20], [21].

In summary, this work identifies a novel combination of molecular targets involved in NOS2/TIMP-1/Akt pro-survival signaling, which influence breast cancer outcome within a specific tumor subpopulation. Importantly, the *in vitro* data provides a basis for identifying agents that may target this merged pathway through TIMP-1 suppression. The identification of downstream targets of NO may be important because the clinical applications of NOS inhibitors have been fraught with cardiovascular complications due to hypotensive response of the patient. Therefore, the elucidation of TIMP-1 as a strong modifier of NO-mediated Akt activation in human tumors circumvents the use of NOS inhibitors and provides an attractive molecular target for patients with elevated pAkt, NOS2, and TIMP-1 protein expression.

## Materials and Methods

### Collection of Tumor Specimens and Survival Information

Paraffin-embedded surgical biopsy specimens and survival information were obtained from 248 breast cancer cases recruited at the University of Maryland Medical Center, Baltimore Veterans Affairs Medical Center, Union Memorial Hospital, Mercy Medical Center and Sinai Hospital in Baltimore, Maryland [56]. The women were recruited between February of 1993 and August of 2003 [28], [29]. All patients were identified through surgery lists and enrolled into the study prior to surgery. They signed a consent form and completed an interviewer-administered questionnaire. Cases were eligible if they resided in the greater Baltimore area at the time of recruitment, were of African-American or Caucasian descent by self-report, had pathologically confirmed breast cancer, were diagnosed with breast cancer within the last 6 months prior to recruitment and had no previous history of breast cancer. Patients were excluded if they were HIV, HCV, or HBV carriers, were IV-drug users, were institutionalized, or were physically or mentally unable to complete and sign the consent and questionnaire forms. Of the eligible patients that were identified through surgery lists, 83% participated in the study. The questionnaire evaluated the medical, reproductive, family and occupational history of the subjects. Additional information including body mass index (BMI), tumor size, node status, disease stage, treatment and survival was obtained from medical records and pathology reports, State of Maryland records, and the Social Security Death and National Death Index. The collection of tumor specimens, survey data, and clinical and pathological information was reviewed and approved by the University of Maryland Institutional Review Board for the participating institutions (UMD protocol # 0298229). IRB approval of this protocol was then obtained at all institutions (Veterans Affairs Medical Center, Union Memorial Hospital, Mercy Medical Center, and Sinai Hospital). The research was also reviewed and approved by the NIH Office of Human Subjects Research (OHSR #2248).

### Histology Evaluation

A pathologist reviewed H&E-stained sections of each tumor specimen, to confirm the presence of tumor and tumor histology. Disease staging was performed according to the tumor-node-metastasis (TNM) system (AJCC/UICC).

### Immunohistochemistry Evaluation

The protein expression profiles of 248 breast tumors have been reported [28], [29]. From these samples, 217 remain available and were evaluated for the impact of TIMP-1 and CD63 protein expression on Akt phosphorylation status and patient survival. All immunohistochemistry reagents were purchased from Biocare Medical (Concord, CA) unless otherwise specified. NOS2, p-caspase-9, and pBAD staining was reported in previous studies [28], [29]. Formalin-fixed and paraffin-embedded 5-µm thickness slides were deparaffinized using three changes of Slidebrite and then rehydrated. The slides were heated at 95°C for 45 minutes in Diva decloaking buffer for antigen retrieval. Endogenous peroxidase was blocked using Peroxidazed I (5 min) followed by treatment with Background Sniper for 10 min. Protein expression was evaluated for TIMP-1 (Dako, Carpinteria, CA), and CD63 (Lab Vision, Fremont, CA). Following incubation in primary antibody, the slides were washed and incubated with MACH 2 Universal HRP-labeled polymer, stained with Betazoid DAB Chromogen kit, and then counterstained with CAT hematoxylin/Tachaís Bluing Solution. Staining specificity was determined with negative and positive control slides. Images of stained sections were taken with an Olympus DP70 Digital Camera System (Olympus, Melville, NY).

### Immunohistochemistry Scoring

A combined score of intensity and distribution was used to categorize the immunohistochemical (IHC) staining for protein expression. Intensity received a score of 0–3 if the staining was negative, weak, moderate, or strong. The distribution received a score of 0–4 if the staining distribution was <10%, 10–30%, >30–50%, >50–80%, or >80% positive cells. A sum score was then divided into four groups as follows: (i) negative = 0–1, (ii) weak = 2–3, (iii) moderate = 4–5, and (iv) strong = 6–7, according to standard protocols for IHC analysis. The ER statuses were scored negative/positive and reported in earlier studies [28], [29].

### Cell Culture

MDA-MB-231 (#HTB-26, ATCC, Manassas, VA) breast cancer cells were grown in RPMI supplemented with 10% heat inactivated FBS and penicillin-streptomycin. Human skin fibroblast (#CCL-110, ATCC, Manassas, VA) were grown in Eagle's MEM supplemented with 10% FBS. Cellular effects of NO were evaluated following exposure to the long lasting NO donor DETA/NO (Dr. Larry Keefer, NCI-Frederick). The donor was prepared at 100 mM stock concentration in 10 mM NaOH to prevent its decomposition. Release of NO from DETA/NO occurred in a pH and temperature dependent manner upon the addition of donor to cell culture media pH 7.4–7.5 (DETA/NO T*_1/2_*∼20 Hr at 37°C). For experiments, the cells were plated and grown overnight (80–90% confluence unless otherwise specified). Cells were treated with DETA/NO for designated times at 37°C in serum-free RPMI (MDA-MB-231) or Eagle's MEM supplemented with 0.5% FBS (fibroblasts). Cell conditioned media of NO-treated fibroblasts was collected and immunoprecipitated for TIMP-1 using an agarose bead conjugated rabbit polyclonal TIMP-1 antibody (Santa Cruz Biotechnology Inc., Santa Cruz CA). The cells were scrape harvested while on ice, centrifuged, and then resuspended in TRIS lysis buffer containing protease and phosphatase inhibitors.

### NOS2 Plasmid Transfection

Cells were transfected with 4 µg pCMV6-XL4 (empty vector) or pCMV6-XL4-human NOS2 (NM_000625) OriGene Technologies, Rockville, MD, USA) by electroporation using the Amaxa Nucleofector kit V (Lonza, Walkersville, MD, USA) and then grown for 48 hours in RPMI+10% FBS supplemented with either L-Arginine (1 mM) or aminoguanidine (5 mM) before Western blot analysis.

### Steady State NO Quantification

Measurement of steady state NO levels was accomplished using a Sievers NO gas analyzer (Boulder, CO). Measurements are representative of steady state NO at 37°C in a 100 mm cell culture dish containing 10 ml serum-free media pH 7.5. Media or buffer (100–200 µl) was injected into a reaction chamber containing 10 mM NaOH to prevent decomposition of unreacted donor. The NO analyzer was continually purged with helium gas to prevent oxidation of NO. Steady state nM concentrations of NO were calculated from the peak area(s) of absolute NO detected and compared to a nitrite (NO_2_^−^) standard curve.

### Suppression of TIMP-1 Protein Translation

Silencing of TIMP-1 protein translation was accomplished using an antisense 25-mer oligo (Gene Tools, Philomath OR) designed specifically to block the AUG translational start site of human TIMP-1 (GenBank #NM_003254: seq 5í-CATGGTGGGTTCTCTGGTGTCTCTC). This oligo complements the sequence from −22 to +3 relative to the initiation codon. MDA-MB-231 cells were plated at a density of 1–2×10^6^ cells per 60 mm tissue culture dish and grown overnight. On the following day 10 µM TIMP-1 antisense or 10 µM control oligo was added to the cells followed by 6 µl, per ml growth media, of Endoporter delivery solution (Gene Tools, Philomath OR) and the cells were incubated for 24 hr at 37°C. The media was then replaced with four ml serum-free RPMI and cell-conditioned media was collected after 24, 48, and 72 hrs. Suppression of secreted TIMP-1 protein lasted through at least 72 hr and was verified by western blotting of the collected cell culture media. Control and TIMP-1 suppressed cells were treated with DETA/NO for 24 hours. The cells were then scrape harvested and cell lysates were evaluated for effects of TIMP-1 suppression on Akt phosphorylation.

### Confocal Microscopy of TIMP-1 Expression in Breast Cancer Cells Treated with NO

MDA-MB-231 cells were seeded at a density of 80,000 cells per well in 8 well Lab-Tek chamber slides (Thermo Scientific, Rochester, NY) and grown overnight. The cells were treated overnight with increasing concentrations of DETA/NO (0, 0.1, 1, 10, 100, 500, and 1000 µM) in serum-free media. The next day, cells were fixed with 1.5% formaldehyde in 200 mM phosphate buffered saline (PBS) for 15 minutes. Following fixation, slides were rinsed with PBS and blocked for 15 minutes with 4% bovine serum albumen (BSA, in PBS). Primary antibodies against rabbit anti-human TIMP-1 and mouse anti-CD63 (Abcam, Cambridge, MA) were diluted in 4% BSA (working dilution of 1∶100 and 1∶250, respectively) in PBS and pre-blocked for 15 minutes. Antibodies were applied to the slides and left to incubate on the cells overnight at 4 degrees in a humidified slide chamber. Slides were washed three times (5 minutes each) with PBS containing 0.01% Tween-20. Slides were then blocked with 4% BSA in PBS for 15 minutes prior to the addition of fluorescent secondary antibodies. Goat anti-rabbit secondary antibodies conjugated to Alexa-488 and goat anti-mouse secondary antibodies conjugated to Alexa 594 were diluted with 4% BSA for a final working concentration of 1∶200 and pre-blocked for 15 minutes. Antibodies were applied to the slides for 1 hour at room temperature in the dark. Slides were washed three times (5 minutes each) with PBS containing 0.01% Tween-20. Nuclei were fluorescently counterstained with 4′,6-diamidino-2-phenylindole (DAPI, 1 mg/ml) in PBS for 5 minutes. The slides were then rinsed in PBS and mounted in Vectashield (Vector Labs, Burlingame, CA).

Confocal microscopy was performed on a Zeiss LSM710 microscope system equipped with a HE/NE laser, Kr/Ar laser, and UV laser. Z-stacks of tumor cells were obtained using 63× oil-immersion objective. Images were displayed as 3D maximum projections images. Image analysis of fluorescent micrographs was performed using ImageJ software [57]. The average fluorescent intensity of pixels corresponding to TIMP-1 expression within cells was sampled using a region of interest (ROI) commonly applied to all cells within the field of view. Background grey values (from areas with no cells) were subtracted from measurements obtained from tumor cells. The mean fluorescent intensity per tumor cell was calculated from 3 randomly chosen fields of view. Mean fluorescent intensity values from tumor cells from 3 images (for each NO treatment condition) were pooled, and the average of the means per condition were shown and compared.

### Identification of TIMP-1 Nitration by Mass Spectrometry

Tryptic peptide data was acquired on a Thermo Finnigan LTQ mass spectrometer equipped with a nano-Electrospray Ionization Source, Finnigan Surveyor MS Pump Plus liquid chromatograph and a Micro AS autosampler (Thermo-Fisher Scientific, Waltham, MA). Six microliters of each tryptic digest was injected onto a nanobore lc column (5 cm×75 um) packed with Biobasic C18 (5 micron particle size, 300 angstrom porosity, New Objective, Inc., Woburn, MA.). Peptides were eluted using a flow rate of 300 nL/minute with precolumn flow splitting and the following linear gradient: 0–80% B in 50 minutes. Solvents A and B consisted of 0.1% formic acid and 90% acetonitrile in 0.1% formic acid, respectively. The LTQ mass spectrometer was operated in a data dependent MS/MS mode using a normalized CID energy of 30%. The electrospray voltage and a heated capillary temperature were 2000 V and 175°C, respectively.

Raw MS/MS data was searched with Bioworks Browser 3.3.1 SP1 utilizing SEQUEST (Thermo Fisher Scientific, Waltham, MA.) against mouse TIMP-1 (UniprotKB accession number P12032) and human TIMP-1 (UniprotKB accession number P01033). For the SEQUEST analysis, the peptide mass tolerance was set at 1.5 Da and the fragment ion tolerance was 0.5 Da. A maximum of 2 missed tryptic cleavages was used in the search. The search criteria included a peptide delta CN 0f 0.1, a peptide SP-Preliminary score of 200, and a peptide Xcorr vs. Charge State of 2.1, 2.3 and 2.9 for 1^+^, 2^+^ and 3^+^ charge states, respectively. Dynamic peptide modifications were search for nitrations (+45 Da) and nitrosylations (+29 Da) for cysteine, tyrosine, serine and threonine.

### Quantitative Real Time PCR

Total RNA was extracted from samples using the TRIzol reagent (Life Technologies, Grand Island, NY) following the manufacturer's protocol. After quantitation and quality check, total RNA was reverse transcribed into cDNA with the RNA to cDNA EcoDry Premix (Clontech, Mountain View, CA). The target gene, TIMP1 and the housekeeping gene, RPL18 primer pairs were designed with the Primer3 primer design engine (http://frodo.wi.mit.edu/primer3/input.htm). Both amplicons were designed to cross exon-exon-junction to minimize non-specific amplification. Target gene's amplification efficiency was verified for similarity to the housekeeping gene prior data analysis. Quantitative real time PCR assay was performed in ABI 7500 system using the standard two steps protocol (95°C 15 s, 60°C 30 s) for 40 cycles. Real time PCR data are acquired and analyzed using Sequence Detection Software v1.4. Delta-Delta-Ct method was adopted for the relative gene expression level calculation.

### Western Blotting

Protein concentrations of cell lysates were determined using the BCA protein assay (Thermo Scientific, Rockford, IL). TIMP-1 was immunoprecipitated using a TIMP-1 rabbit polyclonal antibody conjugated to agarose beads (Santa Cruz Biotechnology Inc., Santa Cruz, CA) and blotted with mouse monoclonal antibodies (TIMP-1, Dako, Carpinteria, CA; CD63 and 3NT, Santa Cruz Biotechnology, Santa Cruz, CA). Heat denatured protein from cell lysates or TIMP-1 IP was electrophoresed on 4–20% SDS-polyacrylamide gels then transferred onto iBLOT stack nitrocellulose membranes (Invitrogen Carlsbad, CA). Transferred protein was blocked for one hr at room temperature in 1% milk in T-TBS and then incubated overnight at 4°C with primary antibodies followed by HRP-conjugated secondary antibodies. The blots were washed and exposed to ECL substrate. Immunoreactive protein was visualized on an Alpha Innotech FluorChem Imager (Cell Biosciences, Santa Clara, CA). Loading controls were verified on stripped blots. For densitometric measurements, total Akt and phosphorylated Akt and BAD protein levels were corrected for local background and quantified using Imagequant Software; pAkt and pBAD were normalized to total Akt. The following antibodies and dilutions were employed: TIMP-1, 1∶2000 (Dako, Carpinteria, CA), total and phospho-specific Akt-ser473, total and phospho-specific PI3k p85, and phospho-specific BAD-ser136 1∶1000 (Cell Signaling, Danvers, MA), HPRT 1∶1000 (Santa Cruz Biotechnology, Inc., Santa Cruz, CA). TIMP-1 levels were also quantified using TIMP-1 ELISA assay (R&D Systems, Minneapolis, MN) according to the manufacturers recommendation.

### Statistical Analysis

Stata/SE 10.1 statistical software (Stata Corp) was used for data analyses of IHC stained tissue. All statistical tests were 2-sided, and an association was considered statistically significant with p<0.05. The Kaplan-Meier method and the log-rank test were used for univariate survival analysis. Survival was determined for the period from the date of hospital admission to the date of the last completed search for death entries in the Social Security Death Index. The mean and median follow-up times for breast cancer survival were 71 months and 68 months, respectively (range, 12–166 months). We obtained death certificates for the deceased case patients and censored all patients whose causes of death were not related to breast cancer. The Cox Proportional-Hazards regression was used for multivariate survival analysis to calculate adjusted hazard ratios. Proportional-Hazards assumptions were verified by log-log plots and with the non-zero slope test of the scaled Schoenfeld residuals. The Spearman rho or Chi^2^ correlation coefficients were calculated for correlation analyses. Multivariate logistic regression was performed to calculate adjusted odds ratios. The following covariates were included in the analyses; age at diagnosis, ER status, and TNM stage. To generate dichotomized data for IHC, scores were divided into moderate to strong versus negative to weak, or into strong versus negative-moderate. Linear regression analysis was employed to determine statistical significance of the relationships between DETA/NO concentration and Akt and BAD phosphorylation status in cell culture experiments. Student T-tests were used to determine significant effects of DETA/NO on TIMP-1 mRNA and protein levels. Results were considered statistically significant with p<0.05.
